# Robust ASV Navigation Through Ground to Water Cross-Domain Deep Reinforcement Learning

**DOI:** 10.3389/frobt.2021.739023

**Published:** 2021-09-20

**Authors:** Reeve Lambert, Jianwen Li, Li-Fan Wu, Nina Mahmoudian

**Affiliations:** ^1^ MS Student, School of Mechanical Engineering, Purdue University, West Lafayette, IN, United States; ^2^ PhD Student, School of Mechanical Engineering, Purdue University, West Lafayette, IN, United States; ^3^ Associate Professor, School of Mechanical Engineering, Purdue University, West Lafayette, IN, United States

**Keywords:** autonomous surface vehicle (ASV), navigation and control, reinforment learning, autonomous vehicle navigation, marine robot navigation, cross-domain deep reinforcement learning

## Abstract

This paper presents a framework to alleviate the Deep Reinforcement Learning (DRL) training data sparsity problem that is present in challenging domains by creating a DRL agent training and vehicle integration methodology. The methodology leverages accessible domains to train an agent to solve navigational problems such as obstacle avoidance and allows the agent to generalize to challenging and inaccessible domains such as those present in marine environments with minimal further training. This is done by integrating a DRL agent at a high level of vehicle control and leveraging existing path planning and proven low-level control methodologies that are utilized in multiple domains. An autonomy package with a tertiary multilevel controller is developed to enable the DRL agent to interface at the prescribed high control level and thus be separated from vehicle dynamics and environmental constraints. An example Deep Q Network (DQN) employing this methodology for obstacle avoidance is trained in a simulated ground environment, and then its ability to generalize across domains is experimentally validated. Experimental validation utilized a simulated water surface environment and real-world deployment of ground and water robotic platforms. This methodology, when used, shows that it is possible to leverage accessible and data rich domains, such as ground, to effectively develop marine DRL agents for use on Autonomous Surface Vehicle (ASV) navigation. This will allow rapid and iterative agent development without the risk of ASV loss, the cost and logistic overhead of marine deployment, and allow landlocked institutions to develop agents for marine applications.

## 1 Introduction

Mobile robots are emerging as powerful tools for scientific exploration in response to societal needs. Robots such as Autonomous Surface Vehicles (ASV), Autonomous Ground Vehicles (AGV), and Unmanned Aerial Vehicles (UAV) have pushed the boundaries of autonomous activity in the water, ground, and air domains respectively. Many applications of autonomous vehicles are mission specific and require retasking, recharging, and reprogramming between missions or activities. For a system to be truly autonomous it must be able to solve navigational problems and avoid obstacles by thinking, planning, and acting and do so across different missions and environments without human intervention. This issue is present across all domains where mobile autonomous robots are deployed.

ASVs are no exception to the rule. While ASVs have been deployed on missions requiring complex navigation such as monitoring coastal ecosystems (Nicholson et al., 2018), exploring coastal waterways (Han et al., 2019), and inland waterways (Karapetyan et al., 2021) they struggle to generalize between these environments and require retasking between deployments in dynamic, rapidly changing, and challenging environments. In general, ASVs have historically struggled to complete missions in such environments like rivers and obstacle ridden littoral waters. For example, an ASV traversing inter-tidal zones will require the ability to sense and rapidly react to obstacles that are exposed with an outgoing tide that it did not experience traversing the area during high-tide. This requires that a vehicle have the ability to robustly process high dimensional and complex environmental data to rapidly make decisions to avoid unknown obstacles that exist in or enter a mission area.

Recent advances in computational power, sensor quality, and algorithms have enabled the application of Deep Learning (DL) to solve the navigation and obstacle avoidance problem in dynamic environments (Giusti et al., 2016; Kahn et al., 2017; Woo and Kim, 2020). A common approach for training DL models is through supervised learning, where a model is fitted to a set of inputs and desired outputs either by classification or regression on predetermined data sets. However, this requires existing data of vehicle navigation. Unlike supervised learning methods, Reinforcement Learning (RL), specifically Deep Reinforcement Learning (DRL), was developed to enable an agent to continuously improve itself through experienced interactions with its environment and thus does not need preexisting datasets.

Large strides in DRL have been realized over the last decade with breakthroughs in an agent’s ability to learn and complete complex tasks such as playing video games (Bellemare et al., 2013), playing board games such as Chess and Go (Silver et al., 2018), and robot body simulations (Todorov et al., 2012). Recently work has shifted towards applying DRL to autonomous vehicle control in real-world situations. The application of deep reinforcement learning to mobile robot navigation and obstacle avoidance has been explored for air (Singla et al., 2021), ground (Lei et al., 2018), and water domains (Cheng and Zhang, 2018; Woo and Kim, 2020; Zhou et al., 2020).

DRL implementations require a significant amount of experiential learning to generalize, which creates a training bottleneck in challenging domains such as water surface and subsea. Access to such domains for training can be exceedingly expensive, logistically challenging, require extensive human presence, be weather dependant, and be potentially dangerous for the vehicle. In contrast, deployment of AGVs and UAVs is comparatively easy, inexpensive, and poses little hazard to vehicles. This has lead to a disparity in data quantity (dataset sizes) and quality (annotated). Many large publicly available datasets are available for terrestrial applications (Cordts et al., 2016; Kellenberger et al., 2018; Perera et al., 2018), while only smaller and more sparse datasets are available for the marine environment (Bovcon et al., 2019; Taipalmaa et al., 2019). Leveraging data rich environments to train DRL agents for data poor environments provides an opportunity to drastically cut developmental times and costs.

Despite the promising application of deep reinforcement learning to autonomous navigation problems, there is limited real-world implementation, especially in the marine environment. The lack of real-world implementation is due to multiple factors, in particular: 1) the significant environmental interaction data required for agent generalization is hard, expensive, and logistically challenging to collect, 2) the constraints of agent training in environments where failure can be detrimental to the vehicle, and 3) implementations can suffer from differences in agent prediction, and vehicle actuation frequencies (Dulac-Arnold et al., 2019).

**FIGURE 1:**
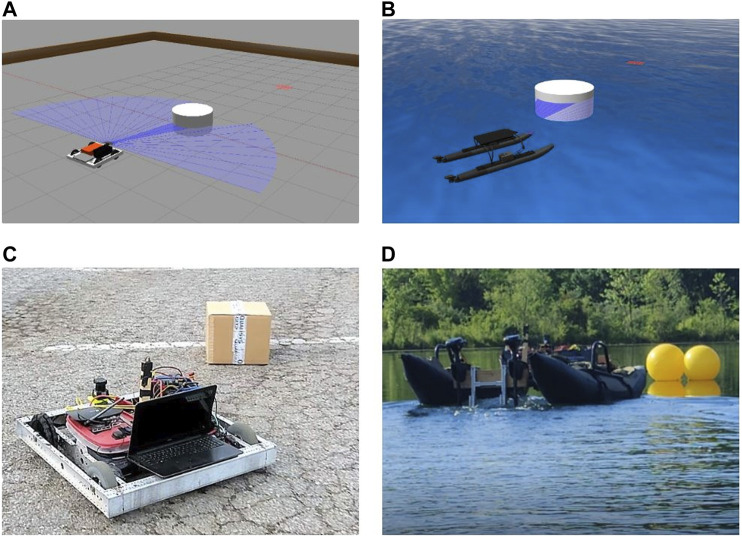
Trained DQN model performing cross-domain obstacle avoidance during **(A)** an AGV simulation, **(B)** ASV simulation, **(C)** AGV real-world mission, and **(D)** ASV real-world mission. All images show execution of the same trained DRL agent.

This paper proposes a methodology for building and implementing well studied DRL agents into mobile robots in such a way that the agent can solve navigation problems and generalize across domains by 1) using an action space that is present in all domains, 2) integrating with a vehicle at a high level through path planning by generating waypoints, and 3) leveraging low-level control for vehicle dynamics and actuation. Following such a methodology that separates the DRL agent from robotic dynamics, an agent can generalize across simulation to real and real to real domain shifts utilizing inexpensive Commercial Off-The-Shelf (COTS) vehicles for initial risky training, can predict at a slower rate than vehicle actuation, and can use accessible domains such as ground to complete training before implementation in hazardous and costly domains such as water. To test the proposed method’s ability to allow cross-domain generalization, a simple Deep Q Network (DQN) is assembled and trained to avoid obstacles on an AGV in a virtual environment and then implemented without alteration or retraining in a simulated water surface environment, on a real-world AGV, and on a real-world ASV for further validation, Figure 1. Both real-world robotic platforms are low-cost COTS systems that operate autonomously using an in-house hardware and software setup developed for multi-domain use.

The success of the proposed methodology will help alleviate domain disparities and decrease the time required for DRL agents to generalize on ASVs by enabling ground and virtual training to be directly applied to the water domain. Knowing that agents trained in simulation and on the ground have a high likelihood of real-world generalization enables confidence in rapid DRL agent iteration on said domains. Lastly, utilizing this methodology landlocked institutions can develop autonomous agents for deployment in marine domains without the need for relocation to coastal areas.

The remainder of this work covers a review of related RL implementations for obstacle avoidance and other related cross-domain ML work in Section 2. Theoretical review of deep neural networks and reinforcement learning methodologies utilized are presented in Section 3. The methodology utilized in DRL agent implementation, the software and hardware package utilized in real-world testing, and a description of the robotic platforms used is detailed in Section 4. The results of agent-testing in simulated and real-world environments are presented in Section 5. Finally a conclusion of the paper and future work are presented in Section 6.

## 2 Related Work

Traditional methods for navigation, obstacle avoidance, and control such as SLAM (Yu et al., 2018), multi-layer controllers (Ferreira et al., 2007), PID control (Caccia et al., 2008), and integral line-of-sight (Fossen et al., 2015) have been extensively studied. While these methods generalize well across domains, customized implementations are required for different vehicles (dynamic differences) and missions (environmental differences). Traditional navigation and control strategies are able to perform adequately in their design scenarios but after customization encounter issues in dynamic environments, incursions by other agents, and upon encountering random obstacles (Gupta, 2013).

Recently there has been increasing effort to solve the issues that traditional controllers experience in dynamic environments with machine and reinforcement learning. Both supervised machine learning and reinforcement learning have typically learned control policies that govern vehicle actuation based on high dimensional sensor data (Mancini et al., 2017; Zhu et al., 2017; Woo and Kim, 2020). These types of ML agents utilize an observation space encompassing the vehicles location, orientation, and its surrounding environment and typically an action space of vehicle actuation commands such as yaw rates and translational speed. Such implementations are considered to be End-to-End.

End-to-End ML and RL implementations think, plan, and act. An agent interfaces with all levels of control. This methodology constrains a trained agent to the specific vehicle it was trained on as it has learned not only how to avoid obstacles and adapt to a changing environment, but also a vehicle’s unique specific dynamics (Cheng and Zhang, 2018; Codevilla et al., 2018; Zhang et al., 2020; Zhou et al., 2020). These implementations would likely struggle generalizing in any cross-vehicle or cross-domain implementation without re-training of the agent.

Some work on DRL implementation has been done in a way that leverages traditional navigational controllers and trains an agent in only the think and plan aspects of autonomous control. Implementations can have the DRL agent switch between path planners to avoid discrete obstacle avoidance scenarios (Woo and Kim, 2020), utilize large action spaces to add 3-D path sub-tasks for tracking (Sun et al., 2020), and utilize higher level Convolution Neural Networks (CNN) to prescribe an observation space for path DRL agents (Yan et al., 2019). These works provide methodologies for indirectly altering the path of a vehicle using typical low-level control, thus providing an agent which could generalize across vehicles. However, they are not made for and are not built to specifically facilitate generalization across domains.

Cross-domain training is not new to the machine learning field. There are many examples of using data gathered in one domain to train an agent designed for implementation in another environment. For example, images and video can be recorded from a car or bike and used in off-line training of convolution neural networks and image recognition nets for a drone (Loquercio et al., 2018). Furthermore, significant work has been done to allow training of agents in a simulated environment with the goal of implementing a trained agent in the real-world (Zhu et al., 2017; Cheng and Zhang, 2018; Osinski et al., 2020; Zhou et al., 2020). However, such an approach typically suffers from the shift between simulation and reality and may still require data gathering in the real-world for full generalization (Zhu et al., 2017).

In addition to cross-domain training, generalizing neural networks between simulation and real-world domains has seen significant work recently under the broad ideology of sim2real. Sim2real implementations do this through training neural networks in randomly generated simulated environments (Tobin et al., 2017), approximating real-world dynamics and textures (Kaspar et al., 2020), or learning high-level control policies (Hong et al., 2018; Müller et al., 2018). Unlike this work, such implementations are typically either end-to-end, or take place in environments with well established environmental dynamics and are used to generate mission paths in a local space rather than augment traditionally globally generated ones.

In this work, a neural network implementation methodology is proposed whereby any high-level dynamic-independent neural agent can be trained in simulation and readily accessible real-world areas (ground and air) and then integrated into existing and proven marine systems (surface and subsea) without altering low level systems. The goal is not to provide another approach to either create realistic simulations that approximate the variances of real-world interaction or generalize neural networks from simulation to the real world through end-to-end visually identified waypoint navigation. Integrating into existing and expensive marine systems to extend their capabilities without training in real and dangerous environment saves time and protects robotic hardware. This work uses the implementation of a DQN trained to avoid obstacles in simulation, on an AGV, and ASV as a case study to prove the viability of the methodology.

## 3 Reinforcement Learning and Deep Q Network Implementation

The problem of sensing and avoiding obstacles can be represented as a Markov Decision Process (MDP). Q-Learning is a form of reinforcement learning that maps the vehicle’s reward through all possible state transitions and actions in such a Markov Decision Process. Deep Q-Learning Networks (DQN) (Mnih et al., 2015) are a form of Q reinforcement learning that utilize Q-Learning and Deep Q-Networks to learn the optimal Q-value function and solve the MDP problem in a stable fashion. Policy-based methods like policy gradient (Silver et al., 2014) solve the MDP problem by finding the optimal policy. In this paper a static obstacle avoiding DQN is used to study and demonstrate the ability of a neural net trained in one domain to be implemented and validated for functionality in another domain. Policy gradient methods require trajectories generated by the current policy, thus are more sample inefficient and are require more training steps to converge and were thus not utilized for implementation in this work. Furthermore, While more advanced DQN implementation strategies such as Double DQN (van Hasselt et al., 2016), Dueling DQN (Wang et al., 2016), and Rainbow (Hessel et al., 2018) exist, a simple DQN was utilized for a proof of concept of cross-domain use on mobile robots.

In the DQN (Mnih et al., 2015), each action (*a*) enacted at an agent’s current state (*s*) has an unknown probability to go to a new state (*s*′) that has associated rewards (positive feedback) and penalties (negative feedback). This positive and negative reinforcement of the action leading to the realized state is called the reward function [*R*(*s*, *a*, *s*′)] and is calculated with respect to the old and new state based on a user defined system that incorporates states to achieve (objectives) and states to avoid (obstacles). A very basic example reward function is shown in Eq. 1 and shows the reward for transitioning from state *s* to state *s*′ through action *a*.

$$R\left(s,a,s^{\prime }\right)=\left\{\begin{array}{c} +50\;\left(\text{Goal reward}\right)\;\\ -100\;\left(\text{Collision penalty}\right)\;\\ R_{\theta }R_{d}\;\left(\text{Position reward}\right)\; \end{array}\right.$$ (1)

*R*_*θ*_ and *R*_*d*_ are the heading reward and distance reward respectively and are defined in Eq. 2. *θ* is the yaw offset from the goal, *D*_*c*_ is the current distance to the goal, and *D*_*g*_ is the initial distance to the goal.

$$R_{\theta }=5\left(1-\frac{2}{\pi }\left|\theta \right|\right)$$ (2)

$$R_{d}=2^{\frac{D_{c}}{D_{g}}}$$ (3)

Each such unique transition and reward of all future transitions is called a Q-value and can be calculated iteratively using the Q-Value Iteration Algorithm in Eq. 4 (Aurelien, 2019).

$$Q_{k+1}\left(s,a\right)\leftarrow \underset{s^{\prime }}{\sum }T\left(s,a,s^{\prime }\right)\left[R\left(s,a,s^{\prime }\right)+\right.\gamma \underset{a^{\prime }}{\max}Q_{k}\left(s^{\prime },a^{\prime }\right)\left.\;\right]\;\text{for all }\left(s^{\prime },a\right)$$ (4)

Where *T*(*s*, *a*, *s*′) is the unknown probability of achieving a new state (*s*′) from an old state (*s*) through action (*a*), *γ* is a discount factor to discount potential future rewards caused by the current state transition, and *a*′ is the optimal action to take after achieving *s*′. By calculating the potential vehicle reward of every possible Q-value, an optimal vehicle policy simply selects the state action pair (*s*, *a*) that deliver the highest possible sum of all discounted future rewards and enacts it. The learning of the unknown probabilities occurs during vehicle deployment as the vehicle either enacts its optimal policy or randomly explores state action pairs and then chooses between the two with an epsilon (*ϵ*) greedy policy.

While Q-learning creates a very stable prediction agent for learning completion, it is impractical in real-world applications with high numbers of state action pairs (Aurelien, 2019). Thus, to improve real-world applications a Deep Neural Network is used as a function approximator [*Q*_*θ*_(*s*, *a*)] to give a Q-value for any input state action pair, such a Deep Neural Network is referred to as a DQN. Thus the goal of agent training is to recursively train the neural network to accurately approximate Q-values.

$$Q_{target}\left(s,a\right)=r+\gamma \cdot \underset{a^{\prime }}{\max}Q_{\theta }\left(s,a\right)$$ (5)

During agent operation the DQN follows the same principles as Q-Learning: it observes the state, chooses an optimal or uniform random action as dictated by *ϵ* greedy policy, transitions to the next state, and receives a reward. The agent stores its interactions with the environment in an experience replay buffer. During training, these interactions—in the form of [*s*, *a*, *s*′, *R*(*s*, *a*, *s*′)]–are randomly extracted in batches and replayed for the DQN, which predicts the reward of the state action pair [*Q*_*θ*_(*s*, *a*)]. With the predicted value and a target reward (Eq. 5, with *r* being recorded rewards) Mean Squared Error (MSE) loss and a RMSprop optimizer the DQN model can be iteratively trained. The better trained the DQN network becomes, the more able it is to select an optimal action that maximizes the sum of all future rewards Eq. 4.

For basic obstacle detection and localization we used LiDAR to provide environmental state sensing, and GPS, digital compass, and IMU to provide vehicle state sensing. The entire state input for our DQN is given in Eq. 6 where *θ*_1_ is the desired heading to the mission objective (as dictated by mission path), *d*_1_ is the distance to the mission objective, *θ*_2_ is the heading to an observed obstacle, *d*_2_ is the distance to said obstacle, and *L*_1_ through *L*_24_ are ranges provided by an on-board LiDAR at 24 linear spaced intervals in the 120° field of view in front of the vehicle. All of the measurements are normalized on [0, 1] before being fed into the DQN neural net.

$$s={\left[\theta_{1},d_{1},\theta_{2},d_{2},L_{1},L_{2},\ldots ,L_{24}\right]}^{T}$$ (6)

$$a={\left[HLT,LT,S,RT,HRT\right]}^{T}$$ (7)

The DQN’s action space is comprised of five discrete actions $\left(a\in R^{5}\right)$. These actions are discrete vehicle commands (Eq. 7) which can be qualified as Hard Left Turn (HLT), Left Turn (LT), Straight (S), Right Turn (RT), and Hard RightTurn (HRT). This small subset of actions are chosen to reduce complexity of the neural network and are interpreted as five vehicle heading augmentations between ± 80° with 40° of separation. These augmentations when combined with vehicle heading are used to define a new waypoint. Larger action spaces will provide smoother paths at the cost of more complex networks. As described earlier action selection is done by selecting the action that has the highest approximated Q-value. While a large continuous action space such as linear and rational rates (a∈R) is common in DRL applications it does not work in this instance as it requires the DRL agent to directly control the agents movements at a low level. Furthermore the base DQN utilized within this work, and other value-based DRL agents, are not capable of outputting continuous actions.

## 4 Methodology

To facilitate cross-domain training and implementation we have created a DRL agent that interfaces with the a given robotic platform at a high level and enables the agent to operate without regard or understanding of the underlying vehicle dynamics. To achieve this, the DRL action space includes path augmentation commands instead of steering and throttle control commands. Simplification of the observation space through sensor state normalization also enables cross-domain generalization. This freedom allows agent abstraction from both vehicle and domain allowing cross-vehicle and cross-domain learning and implementation.

### 4.1 Agent Implementation

**FIGURE 2:**
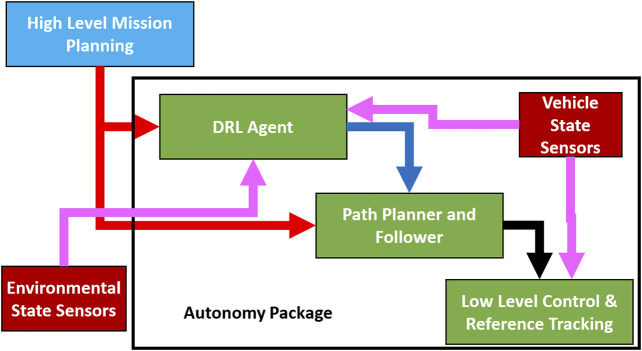
Illustration showing methodology of tertiary controllers integration of the DRL agent and lower level controllers. All processing components are shown in green, autonomy package items are within the black bounding box, mission waypoint flow is shown in red, flow of state information from sensors is shown in magenta, and DRL actions/predictions are shown in blue. Reference headings are shown in black and are based on the DRL action when an action is provided and on the mission path when one is not.

In our methodology, the DRL agent described in Section 3 is implemented as a tertiary level in a multi-level control strategy. This tertiary level serves as both a path augmentation agent for obstacle avoidance and a watchdog for control state switching when an obstacle is sensed in the surrounding environment. As shown in Figure 2, the DRL agent as well as the traditional path planning controller receive commanded mission waypoints (shown in red). While the mission planner always provides a path for the entirety of the mission utilizing a traditional path assembly method, the DRL agent does not provide any input until an obstacle is sensed.

Algorithm 1 Path Augmentation and Waypoint Insertion for Obstacle Avoidance initialization;

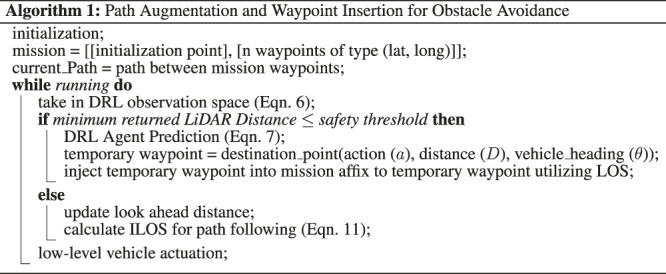

In the event that an obstacle in the environment is sensed in the observation space by the trained DRL agent (Eq. 6), the agent will provide an action from the action space (Eq. 7) which will be given to the path planner. The path planner interprets the DRL agent action into a temporary waypoint which is injected into the mission path. The path planner and follower ceases following the mission path and begins providing low-level control references towards the temporary waypoint, thus navigating away from the nominal path and onto a dynamic path. The temporary waypoint used to build the new path is updated upon every prediction created by the DRL agent (1–2 Hz). This process is shown in Algorithm 1, where the function destination_point is represented by Eq. 8 through Eq. 10, (Veness, 2010). These function will translate DRL actions (Eq. 7) into waypoints. Subscripts of _1_ represent the location of the autonomous agent, subscripts of _2_ represent the new waypoint being created, *λ* represents radians of longitude, *Ψ* represents radians of latitude, *Θ*_1_ is the current heading of the autonomous agent in the global reference frame, *D* is the user set distance away from the boat to create the waypoint (considered 2 m in this work), and *R* is the radius of the earth if represented as a perfect sphere.

$$\begin{array}{ll} \phi & =\left(\left(3-a_{i}\right)\times 40\right)+\Theta_{1}\;\text{where}\;a_{i}\;\text{is the action index with respect to Eqn. }7 \end{array}$$ (8)

$$\begin{array}{ll} \Psi_{2} & =asin(\sin\left(\Psi_{1}\right)\cdot \cos\left(\frac{D}{R}\right)+\cos\left(\psi_{1}\right)\cdot \sin\left(\frac{D}{R}\right)\cdot \cos\left(\phi \right)) \end{array}$$ (9)

$$\begin{array}{ll} \lambda_{2} & =\lambda_{1}+atan2\left(\sin\left(\phi \right)\cdot \sin\left(\frac{D}{R}\right)\cdot \cos\left(\Psi_{1}\right),cos\left(\frac{D}{R}\right)-\sin\left(\Psi_{1}\right)\cdot \sin(\Psi_{2}\right.\left.\;\right)) \end{array}$$ (10)

The DRL agent essentially acts with a carrot and stick strategy, leading the lower control levels where it deems the vehicle should go by redrawing the vehicle path through waypoint creation, but not directly controlling vehicle movement. By focusing on high-level processes the DRL agent leaves real-world dynamic responses to lower level controllers. A PID switching function is implemented on the low level controller to change the aggressiveness of heading error tracking when an obstacle is observed versus not.

The heading reward (Eq. 2) is calculated with respect to the prescribed path, in addition the DRL agent has no insight into the total mission path. This means the DRL is rewarded for following the prescribed path (*θ*_1_ in Eq. 6) when no obstacle is present, which creates a redundant value for vehicle control at the expense of power consumption and heat production. Thus, the predictions of the DRL agent ceases when either no obstacle is perceived or the mission is completed. Once prediction stops, the vehicle re-affixes to the original mission path to resume a nominal trajectory.

The low-level controller receives its reference tracking values from the path planner and has no insight if the reference value is from a nominal mission path or an augmented one. This methodology is implementable in any domain under the assumption that all actuation and path generation is 2-dimensional, as such any 2-D path generation can be utilized with this methodology that allows for dynamic re-planning. This is the case for ground and water vehicles as well as aerial and subsea vehicles operating at constant depth/altitude. The only aspect of this methodology that does not carry between vehicles and domains is the low-level controller. However, such controllers are typically already implemented in commercial autonomous vehicles or can be easily created and tuned.

### 4.2 Autonomy Package

**FIGURE 3:**
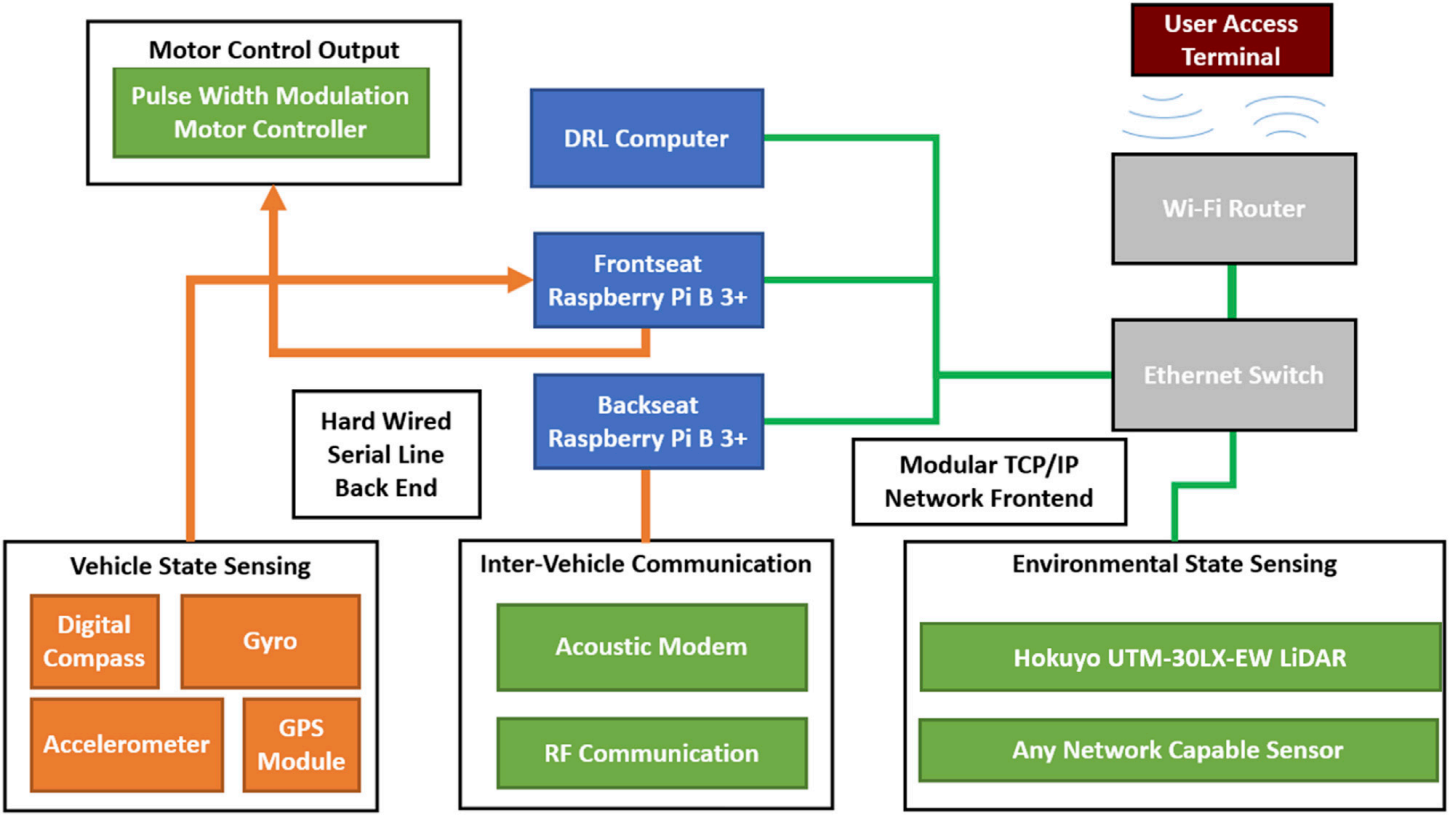
Illustration showing components which comprise the autonomy package. Blue items comprise computational processing items, orange items comprise fixed back-end sensors that are utilized for mid and low-level control, green items are mission specific modular components that can be integrated with computational components over USB (purple lines) or ethernet (green lines), grey items represent network management items that create a local network for distributed computation and sensing.

To facilitate the rapid development of low-cost machine learning capable vehicles across multiple domains a generic and open sourced autonomy package has been developed. The package consists of vehicle and environmental state sensing equipment, communication equipment, and a distributed processing center, Figure 3. The entire package is contained within a splash proof container that can be seamlessly integrated into any AGV or ASV with a 12 V power supply. Implementation in Autonomous Underwater Vehicles or other vehicles is also possible with minimal component packaging modifications.

The distributed processing equipment comprises two low-cost single board computers and a computer capable of running the DQN serves as a computational engine operating together in a distributed processing environment managed by the Robotic Operating System (ROS). Each processing module operates at a different level of vehicle abstraction. The package’s two Raspberry Pi’s operate as a frontseat-backseat duo, while the DRL agent provides prediction and insight into the vehicle’s changing environment. The ROS implementation also allows simulation of the frontseat and backseat by including their respective nodes into a simulated environment.

The backseat (Path Planner and Follower in Figure 2) uses a commanded mission profile (GPS waypoints and times) to generate a path for completion of a delivered mission. This mission path can be a smooth trajectory such as a Dubins path (Dubins, 1957) or straight line between way-points. For this specific work, a line of sight path between waypoints was used for simplicity and construction of missions that force vehicle avoidance around obstacles.

The backseat controller monitors path progression and provides corrected desired bearings to follow the path with an ILOS control strategy (Fossen et al., 2015) as given in Eq. 11.

$$\phi_{D}=\gamma_{P}+tan^{-1}\left(-\frac{1}{\Delta }y_{e}-\hat{\beta }\right)$$ (11)

Where *ϕ*_*D*_ is the desired heading, *γ*_*P*_ is projected heading obtained from the desired path, *y*_*e*_ (cross-track error) is the length of the vector from the vehicle to the prescribed path which intersect the path at a right angle, $\hat{\beta }$ is the estimate of sideslip angle, and Δ is the lookahead distance, which can be set by the user or dynamically updated.

The frontseat (Low-Level Control & Reference Tracking in Figure 2) interfaces with vehicle state sensing and localization using the on-board digital compass, and GPS receiver. The frontseat takes the desired compass bearing and desired velocity provided from the backseat to calculate a desired rotational and linear velocity with two PID controllers that are tuned to the dynamics of the vehicle equipped with the autonomy package.

A graphical representation of autonomy package items, connections, and modularity is shown in Figure 3. The core components and associated cost of the autonomy package are listed in Table 1. The low-cost of the package allows reproduction and repairs to be made quickly and efficiently in the event of a catastrophic failure due to improper agent predictions. This alleviates real-world training risks of vehicle loss. To provide observation space (Eq. 6) values, LiDAR measurements were obtained from a Hokuyo UTM-30LX-EW LiDAR module that was connected to the autonomy package’s network for this series of tests. No other sensors were utilized for these tests, but could be integrated with the autonomy package due to its modular design. Any USB capable or network capable sensor can be integrated without any physical package modifications, only a ROS wrapper is required to make sensor data accessible to the rest of the autonomy package. Thus more complex sensors such as 3D LiDAR, monocular cameras, sonar, and even stereo cameras can be easily incorporated into the architecture to increase the environmental state information available to the DRL agent.

**TABLE 1:** Itemized list of Autonomy Package components.

Item	Number
Raspberry Pi B+	2
NVIDIA Jetson Nano	2
Gumstix Pre-Go GPS Module	1
GPS Patch Antenna	1
HMC6343 Digital Compass	1
Adafruit BNO055 IMU	1
DMC60C Motor Controllers	2
Splash-Proof Enclosure	1
TP-Link N450 WiFi Router	1
TP-Link TL-SG105 Switch	1
5x Ethernet Cables	1
BESTEK 500W Power Inverter	1
LM2596 DC-DC Converter	1
DROK DC-DC Converter	1
6AWG Power quick disconnect cabling	1

The presented methodology for autonomous vehicle control creates a layered architecture with increased levels of abstraction from the vehicles state while also providing layers of interrupt priority ensuring vehicle safety from obstacles while still fulfilling mission priorities. The DRL agent implementation has scope into the entire state of the vehicle and its surrounding environment allowing sensing of obstacles. The frontseat-backseat controllers in the autonomy package have scope of all environmental and mission variables to manage mission objectives and planned paths.

### 4.3 Robotic Platforms

To validate the DRL agent’s ability to generalize across domains two robotic platforms were chosen to utilize the autonomy package to perform autonomous obstacle avoidance dependent missions on the water and ground domains. Both platforms were chosen such that control was achieved through differential steering and had a base platform cost under $900.00 USD. The autonomy package was utilized with both systems to deliver autonomous navigation capability.

**FIGURE 4:**
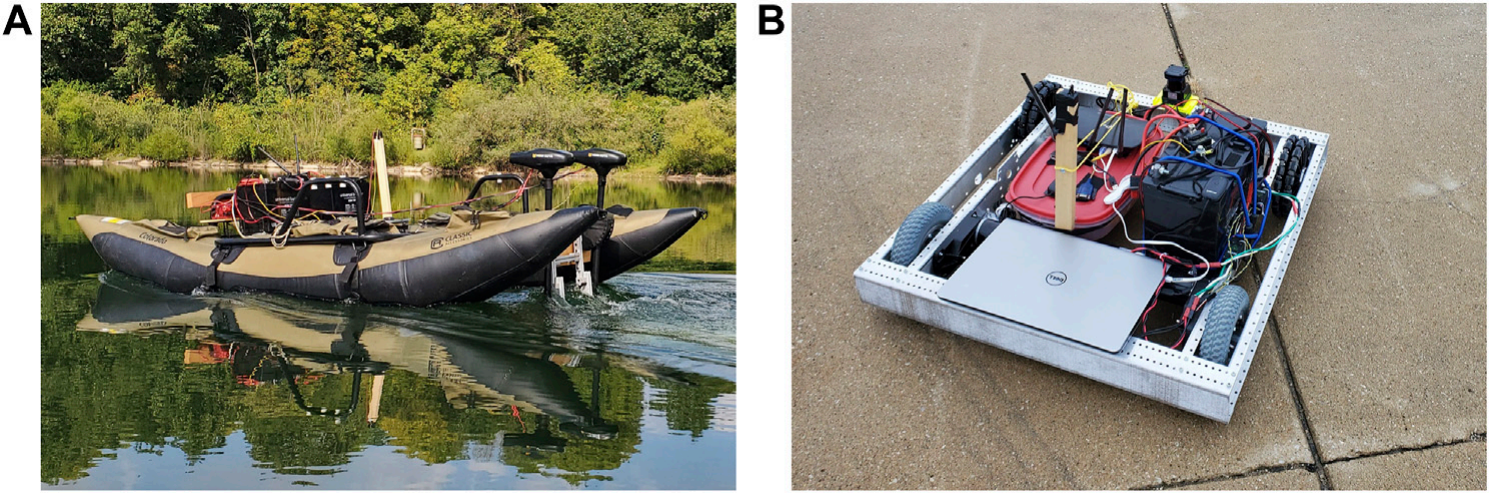
ASV and AGV low-cost COTS vehicles built for use in cross-domain DRL development. Both vehicles utilize the Autonomy Package detailed in Section 4.2.

For the water domain BREAM (Boat for Robotic Engineering and Applied Machine-Learning) (Lambert et al., 2020) was chosen as an ideal platform. BREAM is a low-cost and versatile Autonomous Surface Vehcile (ASV). The low-cost and modular vehicle is driven by two external trawling motors providing differential steering controlled by the Autonomy Package. All components are mounted on a commercial off-the-shelf (COTS) inflatable catamaran hull that provides a total payload capacity of 100 kg in its current configuration. The autonomy package and motors are powered by two 12 V AGM deep cycle marine batteries connected in parallel. Figure 4A shows BREAM with the autonomy package in action. The vehicle has over 60 h of open water operational time completing missions prior to this work.

**FIGURE 5:**
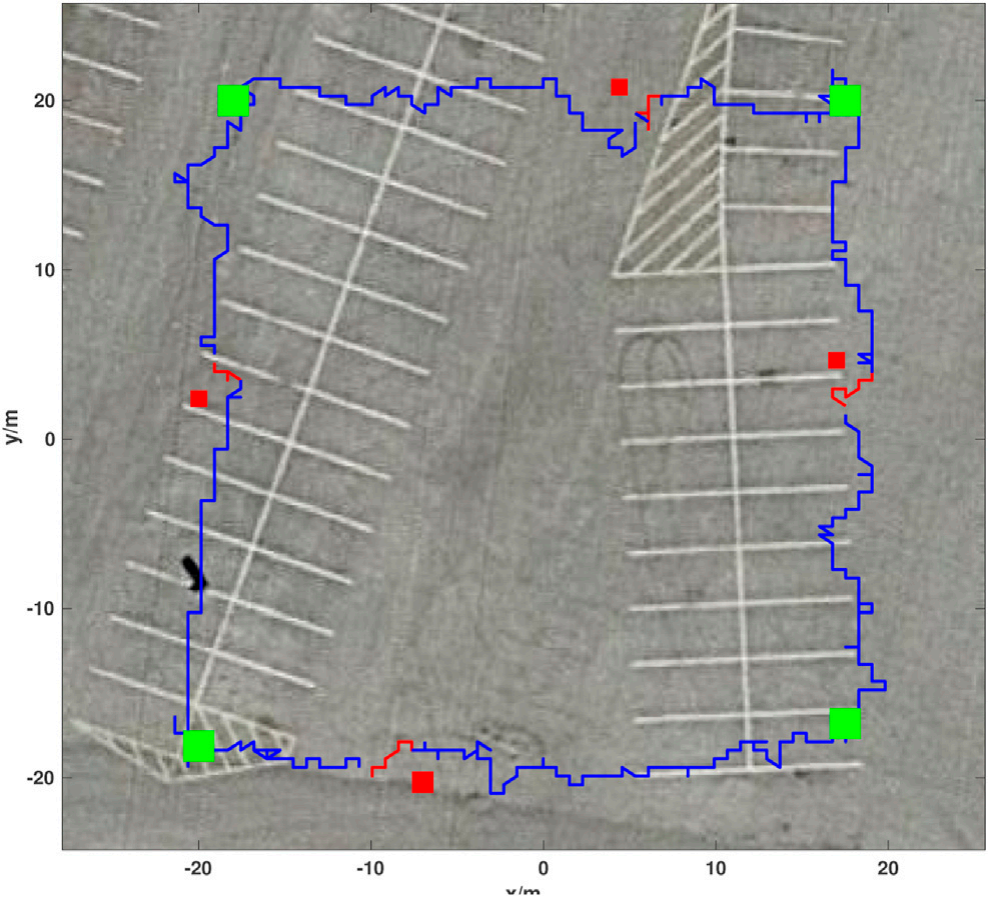
Real-world AGV obstacle avoidance mission showing mission waypoints as green squares, placed obstacles as red squares, DRL agent augmented paths for avoidance in red, and the vehicle trajectory following the mission path in blue. The sample mission shown took 250 s and covered 120 m. The mission trajectory starts at the bottom left mission waypoint and progresses counter-clockwise.

An Autonomous Ground Vehicle (AGV) (Figure 4B) comprised of a COTS chassis and differential drive system was utilized for ground based DRL training and actuation. The base vehicle consists of a chassis, two omni-directional wheels, two pneumatic drive wheels, two brushed DC motors, and a single AGM deep cycle battery. By controlling the vehicle through the autonomy package the vehicle was made fully operational without any modification to the COTS chassis and was able to complete missions autonomously after 15 h of tuning and adjusting low-level control parameters through full deployment in the real-world environment. Further development allowed completion of missions as shown in Figure 5.

In the event that the DRL agent fails to ensure vehicle safety during training/testing both of the high-value low-cost robotic platforms will not put undue financial stress on users. These systems serve as low-risk tools for the rapid development and testing of DRL agents in the real-world. The modularity, inexpensiveness, and simplicity of the autonomy package means that a lost or damaged platform can be easily rebuilt or repaired rapidly for continued testing. Once training is completed and agent confidence is high then the agent can be transplanted to a final platform capable of operation in a specific environment that is desired (off-road, blue water, etc.).

## 5 Experimental Results and Discussion

To test the proposed methodology’s abilities to allow cross-domain generalization a simple DQN is assembled and trained to avoid obstacles on an AGV and then implemented without alteration in a simulated water surface environment and in the real work for further validation. In each case a mission path was constructed by connecting goal waypoints by line-of-sight bearings that connect subsequent waypoints, either in open or closed paths. To quantitatively categorize the amount of deviation from the optimal path (line-of-sight between goal waypoints) cross-track error was utilized as a metric. Initial DRL training was done with a simulated AGV and environment within the Gazebo simulation software. The DRL agent was also implemented on an ASV simulation and on an AGV for operation in urban environments. A comparison between trajectories at different training episodes is utilized to evaluate the DRL agent’s performance. The results for each simulated and real implementation are then discussed.

The DQN model itself is implemented in Python using Tensorflow (Abadi et al., 2016) and Keras. The network itself is contained with a ROS node. Training is done on a desktop computer with a GeForce 2080 GPU and an Intel Core i7-8700 CPU. The AGV testing is done on a laptop with an UHD 620 GPU and an Intel Core i5-5200U CPU. To make the system more compact and protect the computer from water damage, the ASV testing utilized a Nvidia Jetson Nano with a 128-core Maxwell GPU and a Quad-core ARM A57 CPU. The Nano is the end-use computational device for the Autonomy package.

The DQN model has 3 ReLu dense layers, a dropout layer with a dropout rate of 0.2, and a output layer using linear activation function. The learning rate for the DQN is chosen as 2.5 × 10^−4^. The greedy policy used for state exploration *ϵ* starts at 1 and updates every episode. *ϵ* decay rate is set to 0.99 for greater exploration of state spaces. RMSprop is used for optimization. Models will be saved every 10 episodes. An episode is terminated after a collision or if 6,000 steps have occurred (a step takes 0.1 s).

### 5.1 Ground Domain Implementation

To test the autonomy package and train the DQN model, we created a simulation environment using Gazebo. Figure 1 shows the simulated vehicles and obstacles with LiDAR data and target position visualization. The simulated robot receives its position and heading from the gazebo environment. The Gazebo environment contains obstacles and walls that enclose the test area. An AGV model based on the real-world test AGV platform was created using SolidWorks to better simulate the behavior of the AGV’s dynamics. The vehicle consists of an off-the-shelf chassis, four wheels, and two motors which operate in a differential drive configuration. Video of AGV obstacle avoidance can be seen in https://youtu.be/IDkI6rXP1r8.

The obstacles have different shapes and sizes and are placed between mission waypoints. The DQN model is trained during execution of the waypoint navigation tasks. The AGV starts at the first waypoint, and begins moving to all subsequent mission waypoints that are present. If a collision occurs, the AGV model will reinitialize at the origin and another simulation begins.

**FIGURE 6:**
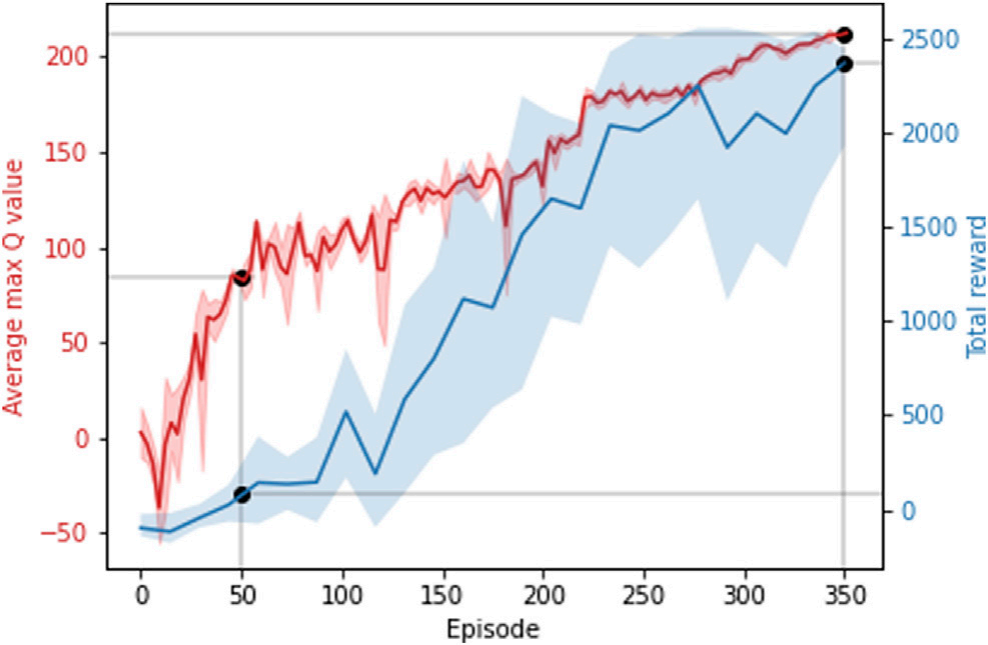
Average max Q-value and total reward across training episodes. Showing the Average max Q-value and the total reward at 50 and 350 episodes.

**FIGURE 7:**
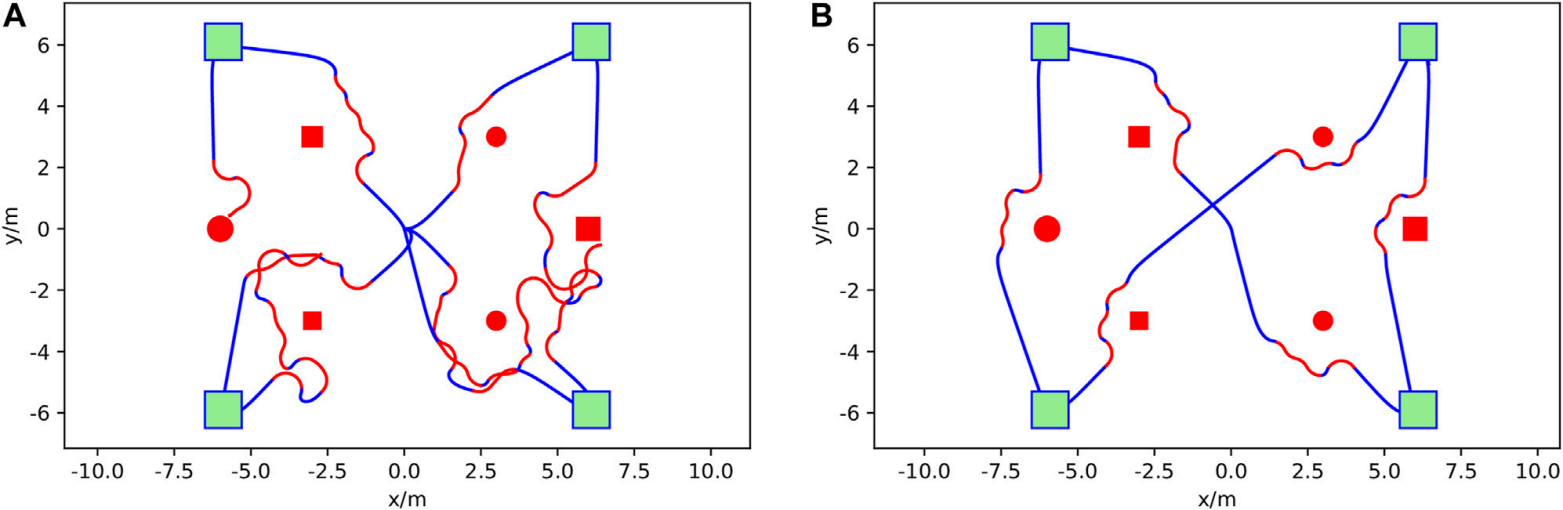
Obstacle avoidance mission comparison at different training episodes in the AGV simulation. **(A)** is at 50 episodes and **(B)** is at 350 episodes. Mission waypoints are shown as green squares, obstacles are shown in red, DRL agent augmented paths for avoidance in red, and the vehicle trajectory following the mission path in blue. The AGV starts from the top right waypoint and then goes to the bottom right, top left, bottom left, and top right in sequence.

It can be seen in Figure 6 that after more than 350 training episodes, the average max Q-value reaches 220 and the total reward reaches approximately 2,400, showing that the average max Q-value and reward increases with respect to training episodes. A reward value of 2,400 contains goal reward values from 1,500 to 2000 (30–40 achieved waypoints) and step reward values of 900–400. A model with such a total reward can avoid obstacles until simulation timeout at 6,000 steps. Figure 7 compares the behavior of the DQN model at different episodic training intervals. At 50 training episodes collisions occurred three times. The average cross-track error was 1.10 m the average deviation across DRL agent avoidance was 1.14 as shown in Table 2, while at 350 episodes the AGV avoided all obstacles and the DQN gave a more optimized trajectory around the obstacles. The average cross-track error was 0.68 m and the average cross-track error across DRL agent avoidance was 0.89 m. When the AGV executed the mission without obstacles, the average cross-track error was 0.16 m. The simulated ground environment formed a baseline model for cross-domain testing.

**TABLE 2:** Cross-track error of different tests. $\bar{y_{e}}$ column lists the average cross-track errors across the entire mission, $\bar{y_{e}}\left(activated\right)$ column lists the average cross-track errors when the DRL agent is activated, and max(y_e_) column lists the max cross-track error observed during the entire test. Tests with no obstacles are not shown within this work.

Test	$\bar{y_{e}}$	$\bar{y_{e}}\left(activated\right)$	**max**(**y**_**e**_)
agv_sim_50_eps	1.10	1.14	3.40
agv_sim_350_eps	0.68	0.89	1.85
agv_sim_no_obstacle	0.16	N/A	0.34
agv_real	1.80	1.93	2.70
agv_real_no_obstacle	0.56	N/A	2.91
asv_sim_with_wind	5.99	7.78	15.59
asv_sim_with_wind_no_obstacle	3.01	N/A	4.98
asv_real_square	1.39	4.88	7.75
asv_real_triangle	1.81	5.66	7.80
asv_real_no_obstacle	0.75	N/A	7.90

To show that the model trained in the simulated environment is valid in the real-world, the trained DQN model was integrated into an experimental AGV platform, Figure 4B and tested. The AGV utilizes the mid and low-level control framework of the autonomy package but a different mission to the simulation. The course utilized for testing is comprised of four waypoints that make up the vertices of a 40 by 40 m^2^.

Four corrugated cardboard boxes were utilized as obstacles for this test. Each boxes is 35 long × 35 wide × 30 cm tall. To better test the model’s performance, obstacles were purposefully placed along the AGV’s prescribed mission path, with one obstacle on each side of the prescribed course during operation to ensure that DRL agent avoidance occurred. Obstacle positions were obtained and overlaid with the AGV’s resulting path, Figure 5. As presented in Table 2, the trajectory has a mean cross-track error of 1.80 m and an average cross-track error from obstacle avoidance of 1.93 m.The trajectory plot shows that the AGV is able to navigate through an environment with static obstacles without collision and find a path to its goals. In contrast, the mean cross-track error is 0.56 when no obstacles were within the mission environment.

### 5.2 Water Domain Implementation

To validate the ability of the DQN model implementation methodology to generalize across different domains, a simulated ASV was tested in a marine environment created in the Virtual RobotX (VRX) simulator (Bingham et al., 2019). To facilitate rapid development of water simulation an open source ASV model of the WAM-V was utilized.

The WAM-V dynamics model was coupled with the DQN agent trained in the AGV simulation and tested on the real-world AGV, as well as the mid and low-level controllers of the autonomy package. The simulation was also given appropriate sensors for environmental and position feedback, specifically a 2-D LiDAR, GPS, and IMU. Gazebo “buoyancy” and “ASV dynamics” plugins were used to simulate the marine environment and dynamic behavior of the vehicle. The simulation used a constant wind disturbance of 5 m per second along the minus *y* axis. Due to the different physical properties of AGV and ASV, larger course and obstacles were setup. All obstacles have a radius of 4 m. The max LiDAR scan range was changed from 3 to 15 m for the ASV to sense and decide whether to react to obstacles and augment the prescribed mission path earlier in the path. However, this did not affect the agent’s trained DQN weights as the observations space values are normalized.

**FIGURE 8:**
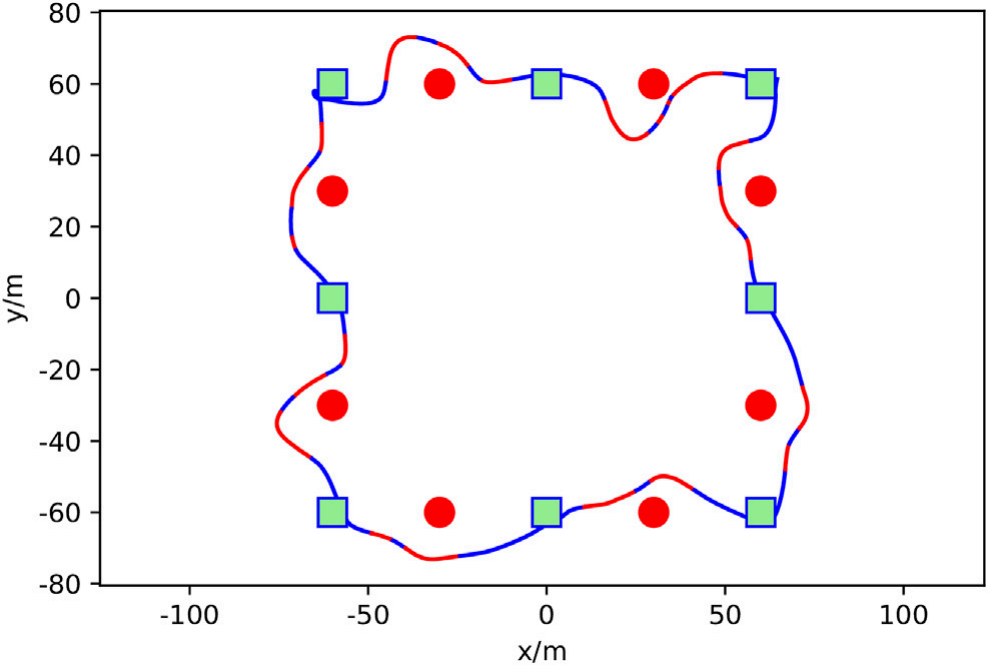
Simulated ASV obstacle avoidance mission showing mission waypoints as green squares, obstacles in red, DRL agent augmented paths for avoidance in red, and the vehicle trajectory following the mission path in blue. The trajectory runs counter-clockwise from the top center mission waypoint.

After loading the agent trained from Section 5.1 and tuning the low level PID controller of the autonomous package to the WAM-V dynamics, the ASV is able to finish the waypoint navigation task without any collisions. The trajectory is shown in Figure 8. As listed in Table 2, the average cross-track error was 5.99 m, a maximum deviation from the optimal path of 15.59 m, and an average cross-track error across DRL agent avoidance was 7.78 m. This validation shows that the DQN agent is able to rapidly generalize across domains for the obstacle avoidance task that it was trained to do with only extremely minimal low level controller changes regarding how rapidly the vehicle alters course to path augmentations. The PID tuning in this case only took approximately 2 h before the ASV was avoiding obstacles as well as the AGV did in real-world implementations. A mission without obstacles was utilized to test the controller’s performance. The average cross-track error was 3.01 m.

To test the DRL agent’s ability to generalize across domains without additional training in a real water environment the BREAM ASV described in Section 4.3 was used. Like the WAM-V model used in the water surface simulation, BREAM is a differentially actuated catamaran ASV. However, unlike the WAM-V (Pandey and Hasegawa, 2015), BREAM is able to be deployed from shore, and is small enough for transportation without a trailer and testing in small lakes or ponds. Furthermore grounding of a WAM-V due to DRL agent failure could prove costly and repairs could be difficult or take a significant amount of time.

**FIGURE 9:**
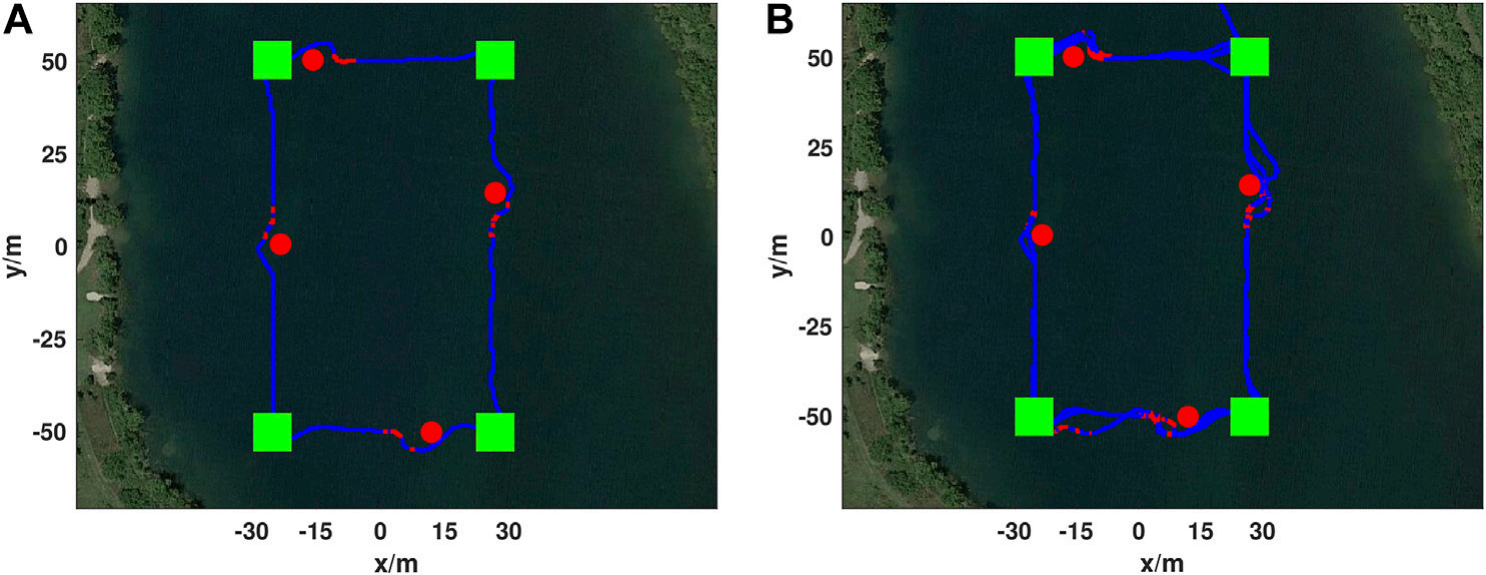
Real-world ASV obstacle avoidance test. Mission waypoints as shown as green squares, placed obstacles as red circles, DRL agent augmented paths in red, and the vehicle trajectory following the mission path in blue. An example trajectory from the test is shown **(A)** and took 252 s and covered 320 m. In total this mission ran for 19 min without collision **(B)** and only stopped due to a human command to stop actuation. The augmented path shown close to the bottom left waypoint (−30, −50) was caused by a passing boat. The mission trajectory starts at (20, 70) and first goes to the top right mission waypoint (30, 50) and progresses counter-clockwise.

To verify obstacle avoidance a mission and associated course was set up at Fairfield Lakes Park in Lafayette, with the resulting course being rectangular in shape with side lengths of 60 and 100 m. This can be seen in Figure 9A. The DRL agent that was trained in the AGV simulation and tested on the real-world AGV and ASV simulation was utilized for this test without any further training on the real-world ASV.

Eight inflatable open water marker buoys were utilized as obstacles for this test, four red and four yellow. Each buoy is spherical with an inflated diameter of 51 cm and is constructed of durable plastic. Each obstacle placed on the course was composed of two such buoys tied together and anchored to the lake bottom with a weight in such a way that minimal slack was present in the anchor line to prevent obstacle drift.

One obstacle was placed on each side of the prescribed course, with the obstacles on the longer sides being set approximately at the midpoint of the side and obstacles on the shorter sides being placed close to the goal waypoint defining one of the course vertexes. The ASV prescribed mission was done so that the ASV traversed in the counter-clockwise direction. The results of the test are shown in Figure 9 with image A showing a single lap around the course, and image B showing 19 min of continual operation around the course. In total two independent tests were done comprising 52 min and 15 s of continuous avoidance, covering over 3.5 km. Video of ASV obstacle avoidance on a rectangular obstacle avoidance test can be seen in https://youtu.be/IDkI6rXP1r8?t=53.

Throughout the test shown in Figure 9 and Table 2 the average cross-track error was 1.39 m, a maximum deviation from the optimal path of 7.75 m, and an average cross-track error across DRL agent avoidance was 4.88 m.

**FIGURE 10:**
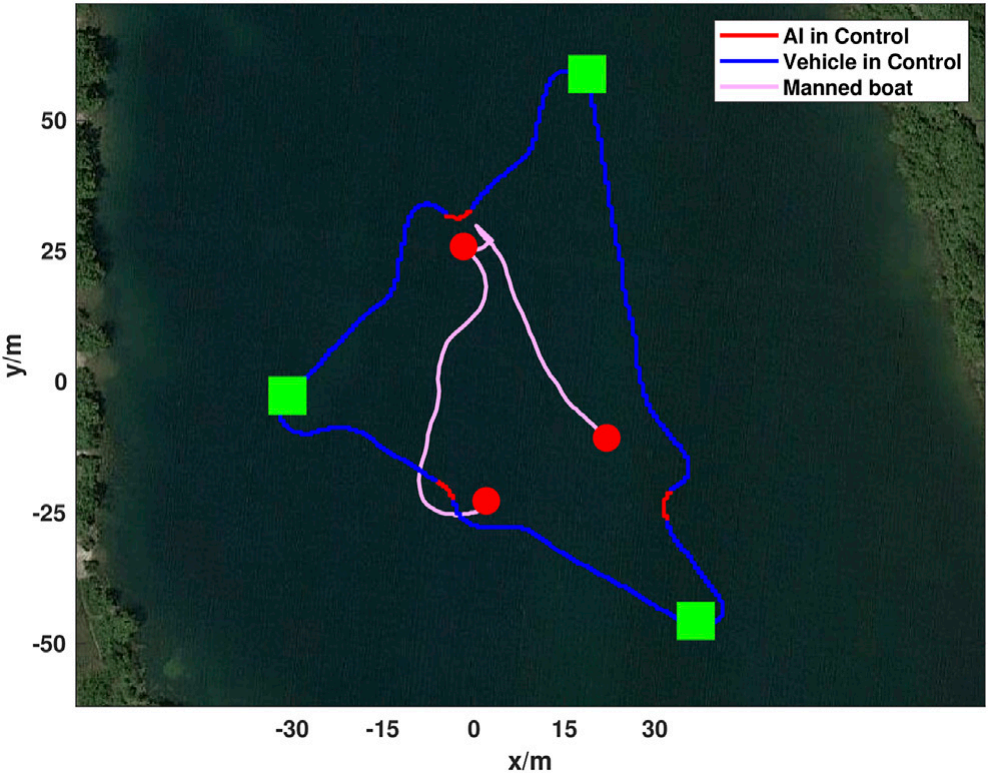
Real-world ASV obstacle avoidance test utilizing a manned boat to inject obstacles into the vehicles path. Mission waypoints are shown in green, ASV DRL agent augmented paths are in red, and the ASV trajectory following the mission path is in blue. The manned boat trajectory is shown in pink and locations where it was first identified as an obstacle by the DRL agent are shown as red circles. Both trajectories start at the bottom left and progress counter-clockwise. The mission shown took 310 s and covered 398 m.

To demonstrate the generalization of the proposed method across dissimilar objects and mission paths a triangular mission was created at Fairfield Lakes, Figure 10. The mission course was run a total of 15 times, however only a single lap is shown for visual clarity. In-place of geometrically simple marker buoys a manned boat was used to intersect the ASV’s path. The manned boat presented an asymmetric surface with both convex and concave surfaces of non-uniform reflectively and color for the ASV to react to. Manned incursions into the vehicle’s path was done to create an unpowered drifting obstacle for avoidance by either crossing or nearly crossing the path. On two occasions during testing contact between vehicles did occur, however this is likely due to the drifting of the manned boat as the DRL agent was not trained to recognize or react to dynamic obstacles. The cross-track error of the mission shown in Figure 10 and Table 2. It was similar to the first ASV mission with a mean cross-track error of 1.81 m, a maximum error of 7.80 m, and an average cross-track error from obstacle path augmentations of 5.66 m. In comparison, the average cross-track error was 0.75 m when the ASV executed a different mission without obstacles. The maximum cross-track error without obstacles was caused by the behaviors and limitations of under-actuated vehicle dynamics when completing a turn-around maneuver. This shows that the effectiveness of the DRL agent is irrespective of obstacle shape or path simplicity.

Throughout testing the largest source of error was the inclination angle and stiffness of the 2-D LiDAR Hokuyo sensor mount on the ASV. With the sensor mounted approximately 30 cm above the waterline the sensor had to be angled properly to allow the 2-D environmental slice to intersect with the water at a proper distance in front of the ASV. Additional problems occurred when the ASV encounter waves and the azimuth of the LiDAR scan relative to the water surface changed. This caused either false positive obstacle readings, or false negative readings depending on the direction of boat pitch. To fix this issue the LiDAR system was mounted on a motorized gimbal to adjust then affix the LiDAR scan azimuth before the start of each test for optimal LiDAR performance. In the future this issue will be solved further by utilizing a 3-D scanning LiDAR.

## 6 Conclusion and Future Work

In this paper, a cross-domain capable obstacle avoidance DRL agent is presented. The agent is implemented utilizing a methodology that facilitates cross-domain training and operation. The methodology includes a tertiary multilevel controller and an autonomy package. The tertiary controller enable the DRL agent to be separated from vehicle dynamics and environmental constraints. The autonomy package provides controllers and electrical hardware to rapidly turn off-the-shelf robots into autonomous DRL agents. The cross-domain obstacle avoidance ability of the methodology was validated with a DQN on an AGV and ASV simulation as well as on a real AGV and ASV. The ASV DRL implementation was also validated with dissimilar obstacles. In each case the DRL agent was able to successfully provide path augmentations to steer robotic platforms clear of obstacles encountered during a mission with minimal path deviation. The results detailed within this work show that the prescribed methodology not only aids in generalizing between ground and water domains but also helps bridge the simulation real-world gap. Thus enabling a reduction in time and cost overhead associated with training in challenging real-world domains.

Future work on this project is extensive. The tests will be expanded to validate the proposed methodology on more advanced DRL models such as Actor-Critic and Double DQN and on higher-dimension sensors such as 3-D LiDAR. These more advanced methods will be used to handle more complex navigational problems such as dynamic obstacles and long-term deployments. The methodology detailed in this work also shows promise in allowing training of complex models in the underwater domain through aerial deployment and overall simulation to real-world transfer. However, further investigation is required to verify the feasibility of transitioning between the domains in more complex settings. In each of these more advanced cases it is improbable that an agent will be able to immediately generalize to its operational domain. A study to determine how well certain observation and action spaces transition between domains and the training required in each case for goal domain generalization is needed in the future. While future work is required, this methodology will serve as a starting point for further cross-domain agent development, which will expand the use of reinforcement learning agents on real-world robotic platforms in challenging environments such as ASVs in the water domain.

## Data Availability

The raw data supporting the conclusion of this article will be made available by the authors, without undue reservation.
